# Do patients’ faces influence General Practitioners’ cancer suspicions? A test of automatic processing of sociodemographic information

**DOI:** 10.1371/journal.pone.0188222

**Published:** 2017-11-22

**Authors:** Rosalind Adam, Roberta Garau, Edwin Amalraj Raja, Benedict Jones, Marie Johnston, Peter Murchie

**Affiliations:** 1 Centre for Academic Primary Care, Institute of Applied Health Sciences, University of Aberdeen, Aberdeen, United Kingdom; 2 Medical Statistics, Institute of Applied Health Sciences, University of Aberdeen, Aberdeen, United Kingdom; 3 Institute of Neuroscience and Psychology, University of Glasgow, Glasgow, United Kingdom; 4 Aberdeen Health Psychology Group, Institute of Applied Health Sciences, University of Aberdeen, Aberdeen, United Kingdom; Universitatsklinikum Tubingen, GERMANY

## Abstract

**Background:**

Delayed cancer diagnosis leads to poorer patient outcomes. During short consultations, General Practitioners (GPs) make quick decisions about likelihood of cancer. Patients’ facial cues are processed rapidly and may influence diagnosis.

**Aim:**

To investigate whether patients’ facial characteristics influence immediate perception of cancer risk by GPs.

**Design and setting:**

Web-based binary forced choice experiment with GPs from Northeast Scotland.

**Method:**

GPs were presented with a series of pairs of face prototypes and asked to quickly select the patient more likely to have cancer. Faces were modified with respect to age, gender, and ethnicity. Choices were analysed using Chi-squared goodness-of-fit statistics with Bonferroni corrections.

**Results:**

Eighty-two GPs participated. GPs were significantly more likely to suspect cancer in older patients. Gender influenced GP cancer suspicion, but this was modified by age: the male face was chosen as more likely to have cancer than the female face for young (72% of GPs;95% CI 61.0–87.0) and middle-aged faces (65.9%; 95% CI 54.7–75.5); but 63.4% (95% CI 52.2–73.3) decided the older female was more likely to have cancer than the older male (p = 0.015). GPs were significantly more likely to suspect cancer in the young Caucasian male (65.9% (95% CI 54.7, 75.5)) compared to the young Asian male (p = 0.004).

**Conclusion:**

GPs’ first impressions about cancer risk are influenced by patient age, gender, and ethnicity. Tackling GP cognitive biases could be a promising way of reducing cancer diagnostic delays, particularly for younger patients.

## Introduction

Delays in cancer diagnosis lead to poorer cancer outcomes [1] and cause patients distress [2]. Longer delays are cited as a reason for poorer cancer outcomes in the UK compared to similarly developed countries [3]. In gate-keeper systems such as the National Health Service (NHS), General Practitioners (GPs) are responsible for most cancer referrals to secondary care. Guidelines support prompt referral of patients presenting with cancer alarm symptoms [4]. However a large proportion of patients subsequently diagnosed with cancer first present with general symptoms which, individually, have low positive predictive values for cancer [5].

A growing body of research suggests that GP intuition plays an important role in their suspicions about cancer [6–9]. Further there is evidence that initial suspicions may influence final diagnosis and referral [10]. It is important to understand factors influencing these early intuitive suspicions. In their experimental study, Kostopoulou et al [10] presented GPs with cancer and non-cancer written scenarios and used a think-aloud and active information search (participants request information in a step-by-step fashion) methodology to explore their decision making. If cancer was articulated as a diagnostic possibility early in the consultation, the patient was significantly more likely to be appropriately referred. While this suggests that early cues within the content of clinical consultations can influence decisions, other non-verbal cues detected on meeting the patient occur earlier than verbal cues and may have an automatic and potent influence.

In face to face human interactions, judgements about an individual’s traits are formed within the first 100 milliseconds of exposure to the face [11]. Medical practitioners will be aware of the benefits of visual first impressions, for example, spotting the sick child in a busy waiting room. Non-verbal cues have also been shown to be clinically important in the context of detecting anxiety and depression [12–14]. Therefore medical decision making is likely to be influenced by both thoughtful, highly informed knowledge systems and by more intuitive, automatic reactions.

A useful approach is to consider the dual-system psychological models of human decision-making. According to these models [15,16] humans use two different cognitive processing methods, referred to as reflective and impulsive processes or as slow thinking and fast thinking. Slow thinking is time-consuming and effortful, involving reflection and deliberation (e.g. considering the pros and cons of action). Fast thinking is automatic and effortless; it is responsible for mental activities such as recognition and response to social cues [15]. Importantly, fast-thinking is error prone and relies upon stereotyping [15,17] while slow thinking involves reasoning [18].

First impressions are important for timeous cancer diagnosis. Previous studies have used written scenarios and vignettes, designed to engage deliberate, slow thinking processes. We hypothesise that the decision making process commences at an even earlier stage, when the GP processes visual information about the patient. We have conducted the first study to explore if visible patient facial characteristics could influence how GPs perceive cancer risk. Incidence of cancer increases with age, and in Scotland it is higher in Caucasians compared to non-Caucasian ethnic groups, and similar by gender [19]. We hypothesise that these visible facial characteristics will influence GP judgement about the relative likelihood of cancer. We further hypothesise that these judgements will be influenced by earlier experience of the epidemiology of cancer: GPs frequent reflective thinking about cancer patients and populations will have translated into automatic responses to epidemiologically relevant cues.

## Materials and methods

### Setting and design

**Fig 1:**
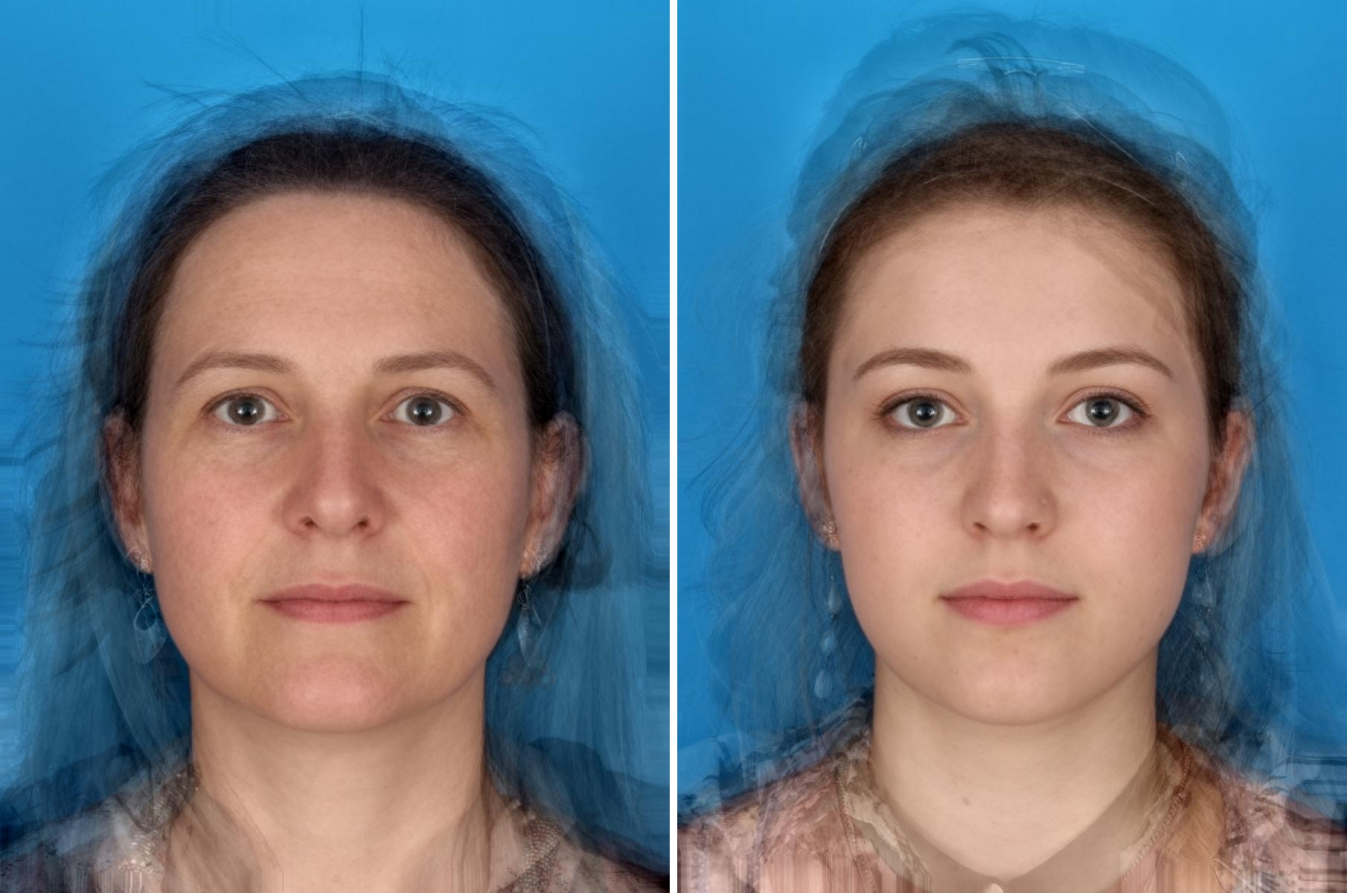
Example of choice set presented to GPs. In this example the comparison is between a middle-aged and a young woman, faces differ in age.

This study took place in Northeast Scotland and featured an online psychological experiment conducted with GPs from Grampian, Orkney, and the Shetland Isles. We designed a web-based binary forced choice experiment. GPs were presented with a choice between two face prototypes (Fig 1). In each pair of face prototypes one key demographic characteristic (age, gender, or ethnicity) was varied. GP respondents were asked to select which of the two face prototypes represented the patient more likely to have cancer and to make their choice quickly based on their first impressions of the face. Early piloting with clinicians indicated that the facial images required introduction. We therefore gave introductions which communicated similarities between the presented pair in brief sentences, as follows: for gender: *“These patients have presented with a three months history of profound fatigue*. *Which one do you think is more likely to have cancer*?*”;* for ethnicity: *“These patients have presented with a four month history of night sweats*. *Which one do you think is more likely to have cancer*?*”;* and for age: “*These patients have presented complaining of unintentionally losing five kilograms in weight*. *Which one do you think is more likely to have cancer*?*”*

### Participants and recruitment

All practice managers in Grampian, Shetland and Orkney were contacted by email through the Scottish Primary Care Research Network and invited to share the study invitation email with GPs in their practice. GPs were also recruited by email via the primary care out of hours (OOH) service. The mailing list includes some GPs who work in local practices, but others who only provide OOH care. Email invitations contained study information, instructions on how to complete the experiment, and a link to the study website. The study was hosted on the ‘faceresearch.org’ website developed by BJ, and the study portal was open for one month. A reminder email was sent before the study end date. There was no remuneration, reward, or financial incentive for participating in the study.

Participating GPs registered on the study website to prevent duplicate study entry, and to allow demographic characteristics to be collected, including gender, age and ethnicity. After completing the experiment, GPs also provided information about job title, years since graduation, years since becoming a GP, practice location, number of doctors in the practice, approximate number of cancers diagnosed, and the characteristics of the last patient diagnosed with cancer. Responses to the experiment were collected anonymously, although GPs could voluntarily provide contact information if they wished to take part in further research.

### Development of the on-line experiment

Each face prototype was manufactured by averaging the shape, colour and textural information from ten individual photographs using professional software developed by the specialist “Facelab” team at the University of Glasgow. Photographs were taken under standardised lighting conditions and with neutral expressions [20]. Face prototypes were created using a previously validated method for capturing age [20].

Photographs from the Facelab database were used to create choice sets in which one variable (either age; gender; or ethnicity) was altered. Female and male prototypes were available, and two non-Caucasian prototypes were possible from the database: young South Asian male and young South Asian female. Photographs were manipulated to create three age variables: young adult, middle-aged adult, and older adult. There were eight available prototypes, allowing for 11 choice sets in the on-line experiment (Table 1). The original images from all 11 choice sets are provided as a supplementary file (S1 Fig).

**Table 1:** Summary of the 11 choice sets in on-line experiment.

Choice sets	Modified variable
Young Caucasian male vs young Caucasian female	gender
Older Caucasian male vs older Caucasian female	gender
Middle-aged Caucasian male vs middle-aged Caucasian female	gender
Young Caucasian male vs young South Asian male	ethnicity
Young Caucasian female vs young South Asian female	ethnicity
Older Caucasian female vs middle-aged Caucasian female	age
Older Caucasian female vs young Caucasian female	age
Young Caucasian female vs middle-aged Caucasian female	age
Older Caucasian male vs middle-aged Caucasian male	age
Older Caucasian male vs young Caucasian male	age
Young Caucasian male vs middle-aged Caucasian male	age

One choice set (of two faces) was presented per page. The face prototypes were presented side by side on a grey background, sized to appear in the centre of the viewer’s screen, and GPs had to click on one of the two prototypes to make a choice. The order of trials and the position of the face in the choice set (left or right), was randomly generated by a computer to avoid order bias. Each choice set appeared only once. GPs were instructed to make their choices quickly, but no time limit was placed on the choice exercise. The length of time each GP took to complete eleven choices was recorded by the software.

### Sample size

Appropriate effect size was difficult to establish due to lack of existing literature. Previous forced choice experiments using face prototypes in studies of human attraction recruited between 120–140 participants [21,22]. However we were interested in the potential impact on clinical decision making and so a small effect on first responses was not of interest. Therefore, following discussion, we powered the study to detect a difference of 15% in preference between choices, and to achieve 80% power and α of 0.05. The necessary sample size was calculated to be 86 GPs [23,24].

### Data handling and statistical analysis

The “faceresearch.org” website allowed administrators to download participant responses as a text file. A full anonymised data set is provided as a supplementary file (S1 Dataset) Data were imported to SPSS version 24.0. Answers to the age (older vs middle aged vs younger), gender (male vs female), and ethnicity (Caucasian vs non-Caucasian) choice sets were recoded as binary numerical variables. Gender of GP, ethnicity of GP, job title and practice location were also assigned numerical values. Age of GP was coded into categories to allow comparison with Scottish GP workforce data [25].

The distribution of responses and 95% Confidence Intervals (CI) were calculated for each choice set related to patient characteristics. Chi-square goodness of fit statistics were used to test the closeness of observed responses to those expected at random. A p-value of less than 0.05 was considered to be statistically significant. However, to account for the effect of multiple testing on Type I error rate, the Bonferroni correction was applied to the p-value [26]. The Bonferroni corrected p-value was 0.05/3 = 0.0167 for the choice set comparing gender, 0.05/2 = 0.025 for the choice set comparing ethnicity, and 0.05/6 = 0.008 for the choice set comparing age.

For multivariate analysis, the GP’s most frequent choice about suspicion of cancer was selected in each category of experiments: gender, age and ethnicity. The most frequent choice was a binary outcome for gender (male vs female) and a categorical outcome for ethnicity (Caucasian, equal choice of Caucasian and South Asian, or South Asian). For the binary outcome, multivariate logistic regressions were performed to assess the influence of GPs’ gender, age, job category, years of service as a GP, number of doctors working in their practice, number of cancers previously diagnosed in their career, and location of their practice on their likelihood of selecting a particular gender (female vs male as reference category). The adjusted odds ratio (aOR) and its 95% Confidence Interval (CI) were used as a measure of strength of association. For the categorical outcome, multinomial logistic analyses were performed to assess the influence of Practice and GP characteristics on the likelihood of selecting a particular ethnicity (Caucasian, equal choice of Caucasian and South Asian or South Asian, with South Asian as the reference category). The adjusted risk ratio (aRR) and its 95% CI from the multinomial model were used as a measure of strength of association. The older face was chosen by almost all GPs (79 out of 82) so associations between GP characteristics and their choices relating to age categories were not examined. Most GPs were Caucasian so GP ethnicity was not included in the multivariate model.

### Ethical considerations

The study was approved by the University of Aberdeen College Ethics Review Board (reference 2015/12/1271) and by NHS Grampian Research and Development (reference 2016GP002). Study information sheets were circulated electronically with invitation emails. At the start of the study exercise each participant was directed to an on-line “informed consent” page, which re-iterated key information about participating in the study, and asked participants to agree or disagree with a written statement of informed consent. Those who agreed could proceed to the on-line study.

## Results

### Study response and participant demographics

Of 665 GPs contacted, 82 GPs participated in the study. Based upon the latest primary care workforce survey [25] we estimated that we reached 545 GPs via medical practices, and 120 GPs through the OOH mailing list, giving an estimated response rate of 13%. GP demographic data are provided in Table 2 and Scottish workforce data [25] are provided for comparison. Of the 82 participating GPs, 47 were female (57.3%), and ages ranged from 30 to 69 years with a mean age 45.9 years and standard deviation 8.7. Of 77 GPs who provided ethnicity data, 76 were Caucasian and one was of Arabian origin. The majority were partners (63.3%) working in urban practices (60.8%).

The mean time taken for GPs to complete all eleven choices was 95 seconds, range 18 to 222 seconds, standard deviation 42.6.

**Table 2:** GP participant demographics.

GP variable		Participating GPs (n = 82)		Scottish GP population [20] (n = 4938)		P value
		Number	%	Number	%	
**Gender**	Male	35	42.7	2096	42.5	1.00
	Female	47	57.3	2842	57.5	
**Age** (Missing 11)	25–34	5	7.0	969	19.7	0.056
	35–44	30	42.3	1505	30.5	
	45–54	22	31.0	1588	32.2	
	55–64	13	18.3	812	16.5	
	≥65	1	1.2	45	1.1	
**Ethnicity** (Missing 5)	Caucasian	76	98.7	No data	No data	n/a
	Non-Caucasian	1	1.3	No data	No data	
**Role** (Missing 3)	Partner	50	63.	3657	73.8	0.106*
	Salaried	16	20.3	692	13.9	
	Retainer	3	3.8	113	2.3	
	Specialty trainee	0	0	492	9.9	
	Locum	10	12.6	No data	No data	
**Practice** **rurality** (Missing 3 participants and 54 missing from Scottish data)	Urban	48	60.8	4231	86.3	**<0.001**
	Rural	31	39.2	674	13.7	

*Retainer, speciality trainees and locum were combined

### Influence of patient’s gender, age, and ethnicity on GP suspicion of cancer

GP choices and results of statistical testing are presented in Table 3. GPs were significantly more likely to suspect cancer in older patients compared to middle aged or younger patients in all choice sets in which age was the modifiable factor: 85.4% (95% CI 75.7–91.5) of GPs chose the middle-aged male over the young male, and 97.6% (95% CI 90.5–99.4) chose the old female over the young female.

**Table 3:** Influence of patient characteristics on GP suspicion of cancer.

Variable of interest	GP Choice Set	GPs making choice in favour of specified variable		GPs making choice in favour of specified variable		p value
Gender		Male		Female		
		Number	Percent (95% CI for percent)	Number	Percent (95% CI for percent)	
	Young male vs young female	59	72.0 (61.0, 80.7)	23	28.0 (19.2, 38.9)	**p<0.001**^**1**^
	Middle age male vs middle age female	54	65.9 (54.7, 75.5)	28	34.1 (24.5, 45.3)	**p = 0.004**^**1**^
	Older male vs older female	30	36.6 (26.7,47.7)	52	63.4 (52.2, 73.3)	**p = 0.015**^**1**^
Age		Older/ middle		Middle/ Younger		
	Young female vs middle-aged female	71	86.6 (77.1, 92.5)	11	13.4 (7.5, 22.8)	**p<0.001**^**2**^
	Middle-aged female vs old female	78	95.1 (87.4, 98.2)	4	4.9 (1.8, 12.5)	**p<0.001**^**2**^
	Young female vs old female	80	97.6 (90.5, 99.4)	2	2.4 (0.6, 9.5)	**p<0.001**^**2**^
	Young male vs middle-aged male	70	85.4 (75.7, 91.5)	12	14.6 (8.4, 24.2)	**p<0.001**^**2**^
	Middle-aged male vs old male	76	92.7 (84.4, 96.7)	6	7.3 (3.3, 15.6)	**p<0.001**^**2**^
	Young male vs old male	73	89.0 (80.0, 94.2)	9	10.9 (2.5, 20.0)	**p<0.001**^**2**^
Ethnicity		Caucasian		Non-Caucasian		
	Young female Caucasian vs young female South Asian	45	54.9 (43.8, 65.5)	37	45.9 34.5, 56.2)	p = 0.377^3^
	Young male Caucasian vs young male South Asian	54	65.9 (54.7, 75.5)	28	34.1 (24.5, 45.3)	**p = 0.004**^**4**^

1 Statistically significant at the Bonferroni corrected p = 0.0167

2 Statistically significant at the Bonferroni corrected p = 0.008

3 Statistically non-significant at the Bonferroni corrected p = 0.025

4 Statistically significant at the Bonferroni corrected p = 0.025

**Fig 2:**
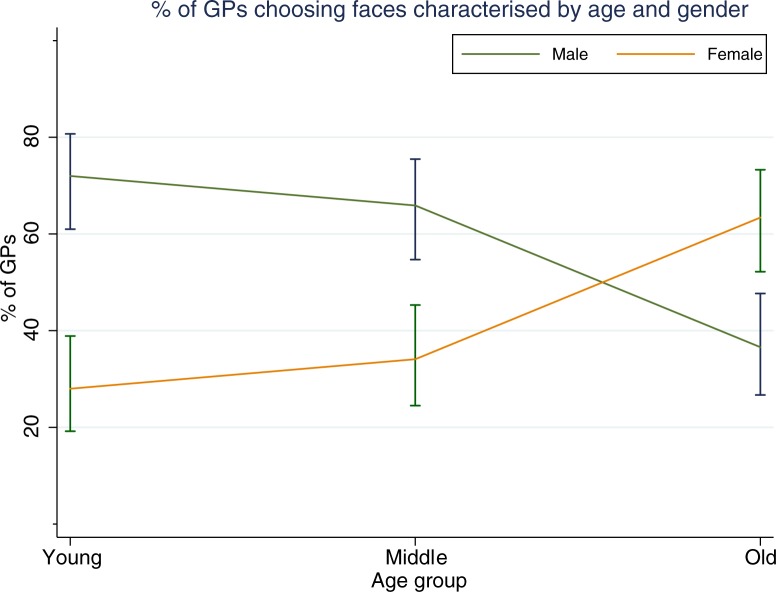
The influence of gender and age on GP choice of face more likely to have cancer.

**Fig 3:**
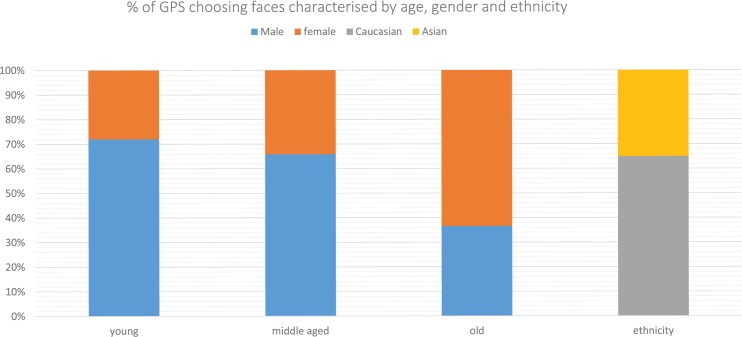
The percentages of GPs choosing age, gender, and ethnicity categories. In the ethnicity comparison (Fig 3), 65.9% (95% CI 54.7–75.5) of GPs decided the young Caucasian male was more likely to have cancer than the young South Asian male (p = 0.004). There was no significant difference in the proportion of GPs choosing between the young Caucasian female and the young South Asian female (54.9% (95% CI 43.8–65.5) chose the young Caucasian female (p = 0.377)).

Patient gender influenced GP suspicion of cancer but this was modified by patient age (Figs 2 and 3). In the choice between young male or young female, 72% of GPs (95% CI 61.0–80.7) decided the young male patient was more likely to have cancer (p<0.001), and 65.9% of GPs (95% CI 54.7–75.5) thought the male (rather than female) patient was more likely to have cancer in the middle age category (p<0.004). In the gender choice set that included older patients, a significantly lower proportion (36.6%; 95% CI 26.7–47.7) of GPs decided that the older male patient was more likely to have cancer than the older female (p<0.015).

### Exploratory analysis of associations between GP characteristics and binary choices

Multivariate regression was carried out (S1 and S2 Tables) to assess for any associations between GP or practice characteristics and the GP’s most frequent choice of a particular gender or ethnicity category (associations were not assessed for age because almost all GPs were more likely to choose older patients). There were no significant associations between GP or practice characteristic on the GP’s likelihood of choosing a particular gender or ethnicity category.

## Discussion

### Summary of results

Patient age, gender and ethnicity significantly influenced GPs’ immediate perception of cancer risk. When presented with a choice between two patients of differing ages, a significant majority of GPs selected the older patient as more likely to have cancer. This was true irrespective of patient gender or ethnicity. Among patients of the same age, gender and ethnicity did influence GPs’ choice regarding the likelihood of cancer. GPs were more likely to select the male patient when presented with young and middle-aged patients, but significantly more likely to select the female patient in the older age category. They also suspected cancer more readily in young Caucasian males compared to young South Asian males. There was no evidence that GPs’ personal characteristics affected their choices.

### Strengths and limitations

To our knowledge, this is a novel study and the first to report on how patients’ demographic characteristics apparent from their faces alone, without verbal cues, influence GPs’ immediate, pre-conscious perception of cancer risk. This is compared to other methods (e.g. talk-aloud or vignette-based) which actively engage conscious thought and reflection.

The cognitive processes involved in reaching a cancer diagnosis have been studied in detail, and involve hypothesis generation, information search, and reasoning [27]. In our study, GPs were asked only to think about cancer risk without alternative diagnoses, and were presented with insufficient information to allow reasoning. The study was not designed to simulate real-life clinical situations, in which GPs consider multiple diagnostic possibilities, and follow initial “suspicions” through to diagnosis. Instead, by deliberately removing the other cognitive processing tasks from the cancer diagnostic process, we have been able to isolate and study the potential role of visual first impressions.

A limitation of our study was the relatively small stimulus set and the limited range of ethnicity choices. The faces presented were averaged from a large pool of faces from the Facelab database and can be considered to be representative of the intended social demographic. Nevertheless, it would be ideal to expand the database for future research in order to create larger and more diverse choice sets.

This study was conducted with a relatively small number of GPs, mainly Caucasian. GPs were from a single geographical area; this probably accounts for the sample not being representative of the Scottish GP population regarding age and rurality of practice. Technically we were hampered by NHS IT security issues, with most NHS computers not allowing respondents to complete the experiment on their workstations. Our statistical power calculation was based on published literature on facial attractiveness, which is not directly comparable to the cancer population. This is the first study of its kind and we would suggest that our preliminary findings are replicated in larger and more diverse samples.

### Comparison with existing literature

Increasing age is a powerful risk factor for cancer [19]. That patients’ age influences GPs cancer suspicion has been reported before in studies in which patient’s age has been manipulated in written vignettes [6–9,28]. While these studies demonstrate that a reasoned, reflective choice is influenced by information about demographic characteristics, our study demonstrates that cancer suspicion may be generated earlier in the consultation process when the only information available is derived from appearance before eliciting verbal information. Collectively, results suggest that GPs’ awareness of the higher probability of cancer in older people greatly influences both their fast, automatic thinking and their slow, reasoning thinking about likelihood of cancer.

Gender is a much less definite risk factor for cancer overall. Scottish incidence rates for all cancers are similar between males and females [19]. Nylenna et al [9] reported a higher prevalence of GP cancer suspicion by GPs in female patients across all age groups, against the epidemiological reality of higher male incidence in Norway at that time. We observed that GP cancer suspicion was significantly heightened by male gender in youth and middle age, but by female gender in older age. This is a striking finding which we cannot readily explain in terms of epidemiological stereotypes but which may nevertheless bias clinical judgements. Doctors’ estimates of the epidemiology of clinical conditions has been found to be influenced by the volume of recent articles referring to the condition in medical journals [29]. When people make judgements about the frequency of events, they are influenced by their ‘availability’, i.e. the number of such events they can bring to mind [30]. There could be some influence from differences in the way that different age groups and genders interact with general practice that may influence availability; for example young men may be more distinct as they consult GPs less frequently [31] and may therefore be more easily remembered, but this is purely speculative.

Regarding ethnicity, a significantly higher proportion of GPs suspected cancer in the young Caucasian male compared to the young Asian male, but interestingly, there was no significant difference between the proportion of GPs choosing between the young female Caucasian and the young female South Asian. In England and Scotland, cancer incidence is considerably higher in Caucasian patients compared to South Asian patients in both genders. As suggested above, male patients may be more distinct and therefore more easily brought to mind, resulting in clearer discrimination of ethnic bias.

It is possible that GPs own characteristics and experience could influence the way they arrive at a cancer suspicion. Our regression model had limited statistical power to explore this, but we found no difference on choices based on GPs’ age and gender and arguably, with the exception of recent significant experiences, these would be the most powerful influencers. Jiwa et al [8] reported that GPs’ with 20 or more years’ experience were significantly more likely to refer patients for suspected cancer, with older GPs significantly more likely to refer urgently. Our study has a different focus since referral decisions are the final culmination of fully conscious clinical decision making processes.

### Implications for research and practice

GPs’ *immediate* pre-conscious impression of a patient’s cancer risk is powerfully influenced by that patient’s age. This has an important implication: younger people with cancer, especially young women, may be at greater risk of a protracted cancer diagnosis from the very moment they present in primary care. However, GPs immediate suspicion of cancer does not appear to be solely based on prior epidemiological probabilities. Some combinations of characteristics appear to have influenced our GPs’ immediate perception of cancer risk more than others. The reasons why deserve further study. Our study has implications for cancer risk prediction tools: GP cognitive biases could arguably be reduced by engaging “slow-thinking” processes such as formal cancer risk assessment tools [32].

Every GP will know of patients diagnosed with cancer unexpectedly following a protracted diagnostic pathway and will be aware that immediate cognitive biases may have played a role. Our study represents an advance in methods to determine how GPs assess cancer risk and is worthy of replication on larger, more diverse samples. A greater understanding of how GPs immediate cancer suspicion is heightened or allayed could inform interventions to reduce the primary care diagnostic interval, with consequent implications for cancer survival.

## Supporting information

S1 Fig Images of every face used to create choice sets in the online experiment. From top left to bottom right these faces represent: young Caucasian female; middle aged Caucasian female; old Caucasian female; young South Asian female; young Caucasian male; middle aged Caucasian male; old Caucasian male; and young South Asian male.
(TIFF)

S1 Dataset The full dataset has been provided on which all calculations have been based. Missing data are assigned "999".
(XLSX)

S1 Table Results of multivariate regression investigating the influence of GP and practice characteristics on GP’s likelihood of choosing a particular gender choice category in the experiment overall. (DOCX)

S2 Table Results of multivariate regression investigating the influence of GP and practice characteristics on GP’s likelihood of choosing a particular ethnicity choice category in the experiment overall. (DOCX)
