# Supplementary figures and images for: Do patients’ faces influence General Practitioners’ cancer suspicions? A test of automatic processing of sociodemographic information
Source: PLoS One. 2017 Nov 22;12(11):e0188222. doi: 10.1371/journal.pone.0188222 (PMC5699847; doi:10.1371/journal.pone.0188222)

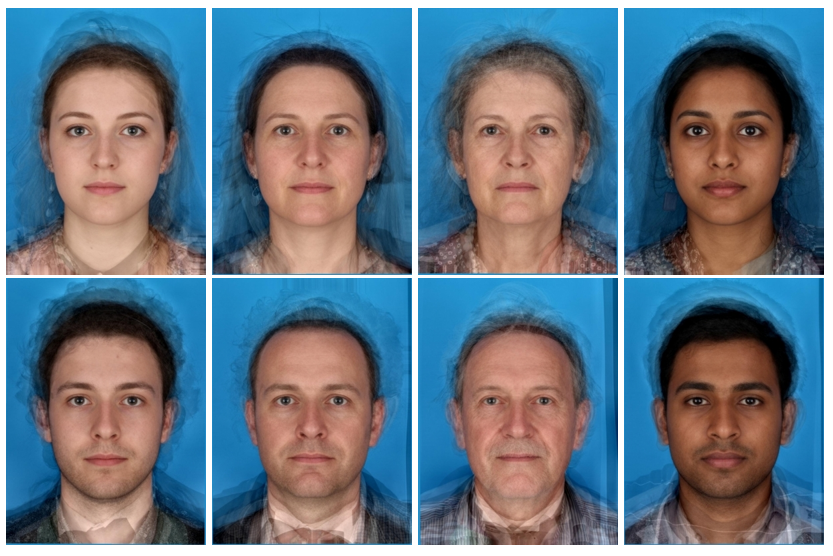

Supplement: S1 Fig — From top left to bottom right these faces represent: young Caucasian female; middle aged Caucasian female; old Caucasian female; young South Asian female; young Caucasian male; middle aged Caucasian male; old Caucasian male; and young South Asian male. (TIFF) [file pone.0188222.s001.tiff]
