# Supplementary material for: Do patients’ faces influence General Practitioners’ cancer suspicions? A test of automatic processing of sociodemographic information
Source: PLoS One. 2017 Nov 22;12(11):e0188222. doi: 10.1371/journal.pone.0188222 (PMC5699847; doi:10.1371/journal.pone.0188222)
Supplement: S1 Table — (DOCX) [file pone.0188222.s003.docx]

S1 Table: Multivariate regression investigating the influence of GP and practice characteristics on GP’s likelihood of choosing a particular (most frequent) gender choice category (male vs female) in the experiment overall.

| **GP Characteristics** | **Number of GPs, N (%)**  **total, n=82** | **Overall choices with respect to patient gender** | | **Adjusted Odds Ratio aOR**  **(95% CI)** |
| --- | --- | --- | --- | --- |
|  |  | **Female**  **(n=29)**  **Number (%)** | **Male**  **(n=53)**  **Number (%)** |  |
| **Gender**  Male  Female | 35 (42.7)  47 (57.3) | 13 (44.8)  16 (55.2) | 22 (41.5)  31 (58.5) | 1.00  0.95 (0.22, 4.06) |
| **Age group (years)**  <45  45-54  55 + | 35 (49.3)  22 (31.0)  14 (19.7) | 13 (59.1)  7 (31.8)  2 (9.1) | 22 (44.9)  15 (30.6)  12 (24.5) | 1.00  7.96 (0.85, 74.39)  50.21 (0.77, 3252.0) |
| **Job title**  Partner  Salaried GP  Retainer or Locum | 50 (63.3)  16 (20.3)  13 (16.5) | 21 (75.0)  3 (10.7)  4 (14.3) | 29 (56.9)  13 (25.5)  9 (17.7) | 1.00  1.64 (0.21, 12.67)  1.76 (0.28, 11.05) |
| **Practice Location**  Rural  Sub urban  Urban | 32 (41.6)  22 (28.6)  23 (29.9) | 12 (44.4)  8 (29.6)  7 (25.9) | 20 (40.0)  14 (28.0)  16 (32.0) | 1.00  1.30 (0.22, 7.65)  1.67 (0.33, 8.31) |
| **Years practicing as a GP** |  | Median (Interquartile Range (IQR) | Median (IQR) | 0.90 (0.74, 1.10) |
|  | 13.5 (10, 22) | 15 (10,20) | 13 (10,25) |  |
| **Number of GPs in current practice** | 7 (5, 11) | 7 (4, 11) | 7 (5, 11) | 0.99 (0.83, 1.17) |
| **Number of cancers diagnosed** | 40 (15, 70) | 45 (17, 70) | 33 (12, 60) | 0.98 (0.95, 1.102) |
