# Supplementary material for: Do patients’ faces influence General Practitioners’ cancer suspicions? A test of automatic processing of sociodemographic information
Source: PLoS One. 2017 Nov 22;12(11):e0188222. doi: 10.1371/journal.pone.0188222 (PMC5699847; doi:10.1371/journal.pone.0188222)
Supplement: S2 Table — (DOCX) [file pone.0188222.s004.docx]

S2 Table: Multivariate regression investigating the influence of GP and practice characteristics on GP’s likelihood of choosing a particular (most frequent) ethnicity choice category in the experiment overall.

| **GP Characteristics** | **Number of GPs,**  **Number (N) (%)** | **Overall choices with respect to patient ethnicity** | | | **Caucasian**  **vs South Asian**  **Adjusted Risk Ratio**  **(95% CI)** | **Equal frequency of Caucasian/South Asian choices vs South Asian**  **Adjusted Risk Ratio**  **(95% CI)** |
| --- | --- | --- | --- | --- | --- | --- |
|  |  | **South Asian**  **(n=14)**  **(N) (%)** | **Caucasian**  **(n=31)**  **N (%)** | **Equal frequency of choices for both South Asian and Caucasian categories**  **N (%)** |  |  |
| **Gender**  Male Female | 35 (42.7)  47 (57.3) | 7 (50.0)  7 (50.0) | 13 (41.9)  18 (58.1) | 15 (40.5)  22 (59.4) | 1.00  7.18 (0.79,64.9) | 1.00  4.50(0.66, 31.05) |
| **Age group (years)**  <45  45-54  55 + | 35 (49.3)  22 (31.0)  14 (19.7) | 6 (42.9)  6 (42.9)  2 (14.3) | 12 (48.0)  6 (24.0)  7 (28.0) | 17 (53.1)  10 (31.3)  5 (15.6) | 1.00  3.01 (0.19, 46.56)  183.9 (0.56, 60629.0 | 1.00  0.92 (0.8, 11.36)  9.00 (0.04, 1908.1) |
| **Job title**  Partner  Salaried  Retainer/locum | 50 (63.3)  16 (20.3)  13 (16.5) | 8 (61.5)  3 (23.1)  2 (15.4) | 20 (66.7)  4 (13.3)  6 (20.0) | 22 (61.1)  9 (25.0)  5 (13.9) | 1.00  0.21 (0.01, 2.99)  0.97 (0.08, 11.56) | 1.00  0.25 (0.02, 2.99)  0.48 (0.04, 5.66) |
| **Practice type**  Rural Suburban Urban | 32 (41.6)  22 (28.6)  23 (29.9) | 7 (53.9)  2 (15.4)  4 (30.8) | 10 (32.3)  9 (29.0)  12 (38.7) | 15 (45.5)  11 (33.3)  7 (21.2) | 1.00  1.80 (0.11, 30.93)  2.60 (0.30, 22.80) | 1.00  4.32 (0.38, 48.78)  1.65 (0.22, 12.27) |
| **Years as GP** |  | Median (Interquartile Range (IQR)) | Median (IQR) | Median (IQR) | 0.72 (0.54, 0.95) | 0.84 (0.65, 1.08) |
|  | 13.5 (10, 22) | 15 (12, 24) | 13 (10, 22) | 13 (10, 21) |  |  |
| **Number of GPs in the practice** | 7 (5, 11) | 10 (7, 11) | 6 (4, 12) | 6 (4, 11) | 0.78 (0.59, 1.01) | 0.78 (0.61, 1.00) |
| **Number of cancer patients diagnosed** | 40 (15, 70) | 35 (14, 50) | 33 (10, 70) | 40 (20, 75) | 1.05 (0.99, 1.11) | 1.04 (0.98, 1.09) |
